# High-resolution vessel wall imaging-driven radiomic analysis for the precision prediction of intracranial aneurysm rupture risk: a promising approach

**DOI:** 10.3389/fnins.2025.1581373

**Published:** 2025-04-22

**Authors:** Wenqing Yuan, Shuangyan Jiang, Zihang Wang, Chang Yan, Yongxiang Jiang, Dajing Guo, Ting Chen

**Affiliations:** ^1^Department of Radiology, The Second Affiliated Hospital of Chongqing Medical University, Chongqing, China; ^2^Chongqing Medical University, Chongqing, China; ^3^Department of Neurosurgery, The Second Affiliated Hospital of Chongqing Medical University, Chongqing, China

**Keywords:** radiomics, intracranial aneurysm, high-resolution vessel wall imaging, machine learning, rupture

## Abstract

**Objective:**

This study aimed to extract the radiomic features of intracranial aneurysm (IA) and parent artery (PA) walls from high-resolution vessel wall imaging (HR-VWI) images and construct and validate machine learning (ML) predictive models by comparing them with the radiomics score (Rad-score).

**Methods:**

In this study, 356 IAs from 306 patients were retrospectively analyzed at Yuzhong Center and randomly divided into training and test cohorts in an 8:2 ratio. Additionally, 66 IAs from 58 patients were used at Jiangnan Center to validate the predictive model. Radiomic features of the IA and PA walls were extracted from the contrast-enhanced HR-VWI images. Univariate and least absolute shrinkage and selection operator (LASSO) regression analyses were performed on the training cohort features to identify optimal rupture-associated features. The Rad-score model was constructed by calculating the total score derived from the weighted sum of optimal radiomic features, and three ML models were built using the XGBoost, LightGBM, and CART algorithms, and evaluated using both the test and external validation cohorts.

**Results:**

Eight optimal IA wall features and four PA wall features were identified. The Rad-score model demonstrated an area under the curve (AUC) of 0.858, 0.800, and 0.770 for the training, test, and external validation cohorts, respectively. Among the three ML models, the XGBoost model performed best across all cohorts, with AUC values of 0.983, 0.891, and 0.864, respectively. Compared to the Rad-score model, the XGBoost model exhibited superior AUC values (*p* < 0.05), better calibration curve Brier scores, and greater net clinical benefit.

**Conclusion:**

The radiomic features extracted from HR-VWI images demonstrated robust predictive utility for IA rupture risk in both the Rad-score and ML models. The XGBoost-based ML model outperformed the Rad-score model in efficacy and performance, and proved to be a noninvasive, efficient, and accurate tool for identifying high-risk IA patients.

## 1 Introduction

Intracranial aneurysms (IAs), affecting 1–3% of adults, are a leading cause of aneurysmal subarachnoid hemorrhage (Etminan et al., 2019; Sauvigny et al., 2020; Tjoumakaris et al., 2024). Initial rupture carries a 30–40% mortality rate, while re-bleeding escalates this to 70–80%. Overall, IA rupture leads to disability or death in 25–50% of cases (Malhotra et al., 2018; Frączek et al., 2024).Early detection and accurate rupture risk assessment are crucial for effective clinical management.

Cerebrovascular imaging techniques are pivotal for diagnosing, treating, and preventing IAs. Digital subtraction angiography (DSA) remains the gold standard for morphological assessment of IAs due to its unparalleled spatial and temporal resolution (Veeturi et al., 2024). However, its invasive nature, associated procedural risks, and limited ability to evaluate vessel wall pathophysiology restrict its utility in routine clinical surveillance. Computed tomography angiography (CTA) and magnetic resonance angiography (MRA) provide non-invasive alternatives but are constrained by their focus on luminal morphology and insufficient resolution for detailed wall characterization. In contrast, high-resolution vessel wall imaging (HR-VWI) offers a paradigm shift by enabling non-invasive, three-dimensional visualization of both the IA and parent artery (PA) walls, shifting the assessment focus from the lumen to the vessel wall (Larsen et al., 2018; Lehman et al., 2018; Samaniego et al., 2019; Semin et al., 2023). Moreover, HR-VWI reveals arterial wall enhancement (AWE) as a critical marker of inflammatory activity, offering significant value for rupture risk assessment (Lauric et al., 2022). This capability positions HR-VWI as a critical tool for advancing precision in IA risk stratification.

However, the interpretation of HR-VWI images remains subjective, highlighting the need for more objective quantitative assessment methods (Veeturi et al., 2022; Dier et al., 2024a). Radiomics addresses this by extracting extensive image features to achieve comprehensive quantitative characterization of lesions. Recent studies have demonstrated the application of radiomics in quantifying aneurysm wall enhancement (AWE) heterogeneity (Veeturi et al., 2023, 2025; Dier et al., 2024b) and validated that radiomic-based models outperform traditional methods in predicting IA rupture risk (Tong et al., 2021; Lauric et al., 2022). Despite these advances, more robust and comprehensive HR-VWI-based models are still required for reliable IA rupture risk assessment.

Therefore, this study aims to develop a more objective predictive model by integrating radiomic features from HR-VWI-based IA walls and PA walls, combined with radiomics scores (Rad-scores) and machine learning algorithms, to enable more accurate assessment of IA rupture risk.

## 2 Materials and methods

### 2.1 Patient selection

This study was approved by the Institutional Review Board. Written informed consent was obtained according to ethical guidelines (Ethics No. Colum Review No. 194). Clinical and imaging data were comprehensively collected and statistically analyzed from patients diagnosed with IAs at the Yuzhong and Jiangnan centers between January 2020 and July 2024.

Patients with IAs were included, with HR-VWI images of the IAs and the PAs. The included patients can be categorized into the following groups: (1) unruptured aneurysm patients: individuals in whom aneurysms were incidentally discovered through imaging studies; (2) ruptured aneurysm patients: patients with a confirmed aneurysm accompanied by subarachnoid hemorrhage. The exclusion criteria were as follows: (1) incomplete HR-VWI datasets; (2) non-aneurysmal SAH or coexisting cerebrovascular diseases; (3) treated IAs; (4) unclear responsible artery for rupture in multiple IAs; (5) poor HR-VWI quality.

**Figure 1:**
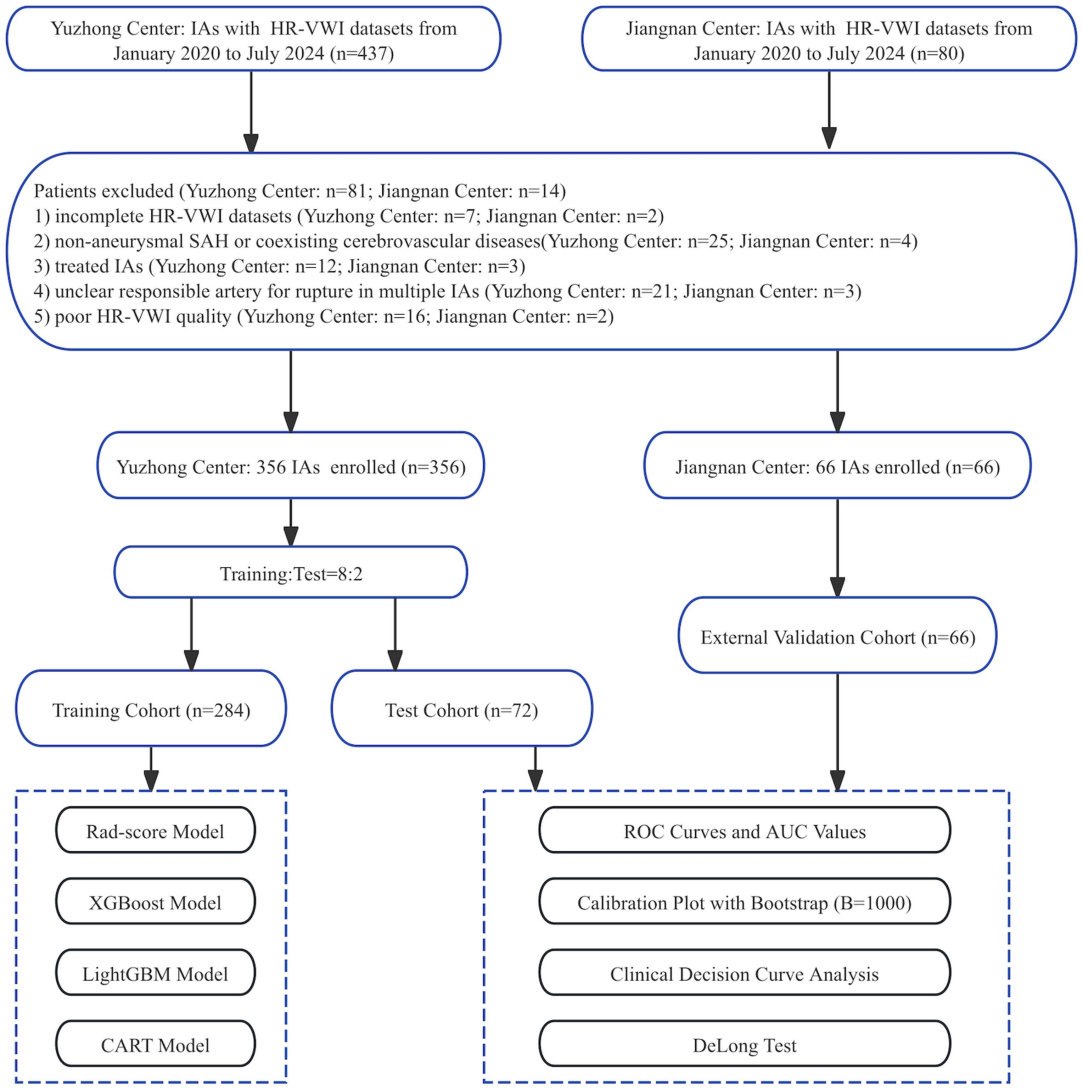
Screening workflow chart.

**Figure 2:**
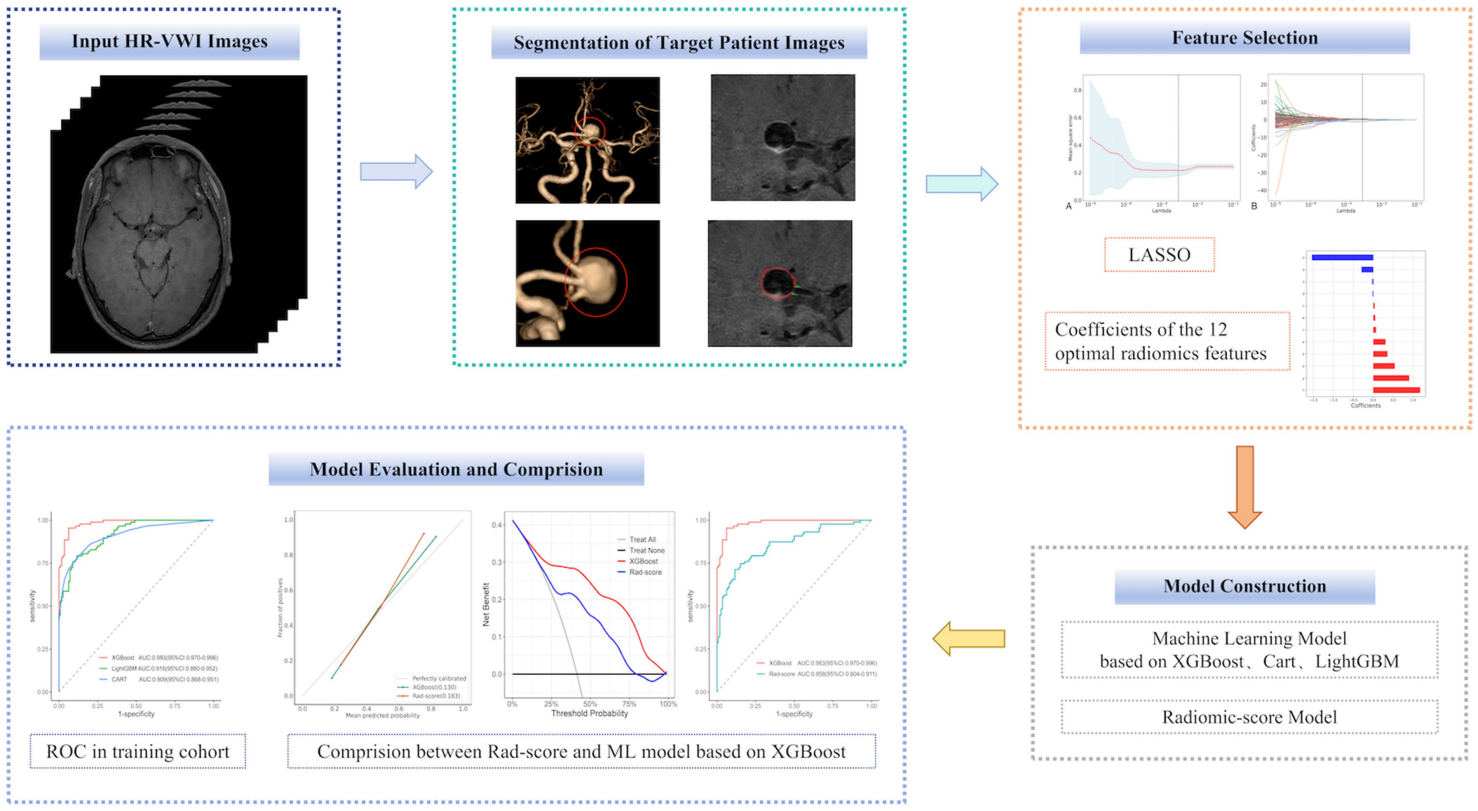
Overall ML and Rad-score modeling framework.

In this study, 356 ruptured and unruptured IAs from 306 patients were enrolled at the Yuzhong Center and randomly categorized into training and test cohorts in an 8:2 ratio. The cohorts were used for radiomic feature extraction. Specifically, 284 (80%) IAs were designated as the training cohort to develop the Rad-score and three ML models, while 72 (20%) IAs constituted the test cohort. Furthermore, 66 IAs from 58 patients were enrolled at Jiangnan Center for model validation. The screening workflow chart and overall ML and Rad-score modeling framework are shown in Figures 1, 2.

### 2.2 Patient image acquisition

Magnetic resonance imaging (MRI) was performed on all subjects using Ingenia CX 3.0 T MRI scanner equipped with a 32-channel head coil (Philips, Best, Netherlands). The imaging protocol commenced with three-dimensional (3D) time-of-flight (TOF) MRA for IA localization, followed by axial 3D T1-weighted isotropic turbo spin-echo acquisition HR-VWI sequences focused on the PAs. A contrast agent, methylammonium gadopentetate (0.1 mmol/kg, Gd-DOTA, Jiangsu, China), was administered via manual injection into the antecubital vein. HR-VWI was repeated 5 min post-injection to acquire contrast-enhanced HR-VWI (CE-HR-VWI) images over identical anatomical ranges. The detailed scanning setup parameters are presented in Supplementary Table S1.

### 2.3 Segmentation of target patient images

**Figure 3:**
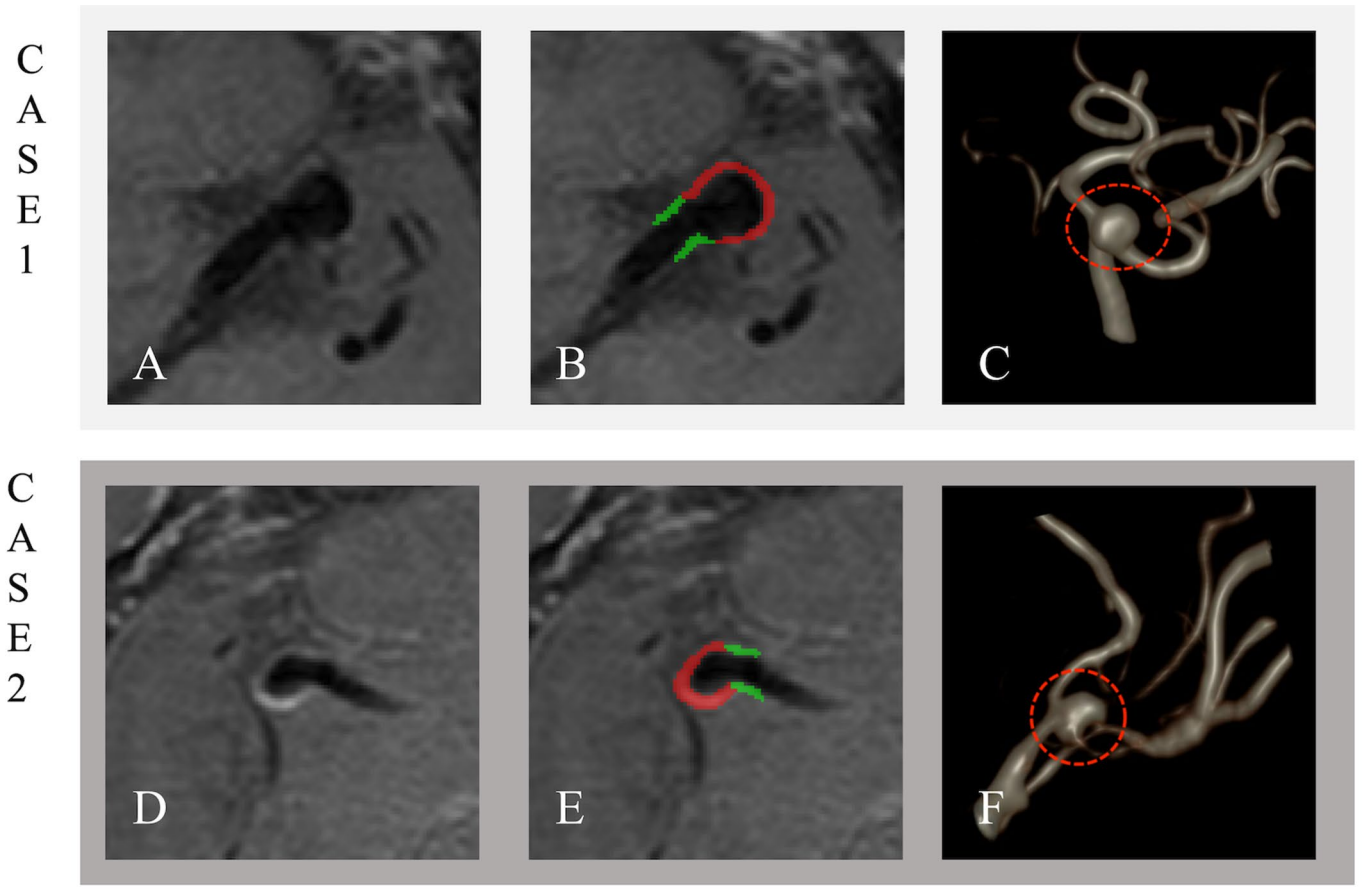
Unruptured and ruptured aneurysms in Cases 1 and 2. Case 1: A 57-year-old male with an unruptured 5.3 × 4.4 mm aneurysm on the left MCA M1 segment. Case 2: A 49-year-old male with a ruptured 7.0 × 3.4 mm aneurysm on the right MCA M1 segment. The Panel shows original imaging **(A,D)**, outlined aneurysms **(B,E)**, and volume-rendered reconstructions **(C,F)**. The IA wall is in red, and the PA wall is in green.

The original CE-HR-VWI images were imported into the ITK-SNAP software (version 4.0.2) in DICOM format. The IA wall was manually delineated on the slice with the maximum IA diameter, while the PA wall was manually traced within 3 mm on both sides of the aneurysm neck. The process of segmenting target patient images is illustrated in Figure 3.

### 2.4 Image preprocessing and feature stability analysis

All images were preprocessed to ensure consistency. The voxel dimensions of CE-HR-VWI sequences (0.6 × 0.6 × 0.6 mm^3^) were isotropic. Image intensity normalization was performed using Z-score normalization (mean = 0, standard deviation = 1) to mitigate scanner variability. Addtionally, the gray-level intensity values were discretized into 64 equidistant bins to reduce noise and enhance the robustness of the radiomic features. Subsequently, the radiomic features were then automatically extracted from the IA walls and PA wall ROIs using the PyRadiomics platform (version 3.0.1).

To evaluate the stability of radiomic features, a reproducibility analysis was conducted on a randomly selected subset of 100 patients from the training cohort. Specifically, one month apart, the aneurysm wall and vessel wall ROIs of each patient were manually re-segmented using the method mentioned above by the same radiologist (with over 10 years of experience in neuroimaging diagnosis) and another radiologist (with over 15 years of experience in neuroimaging diagnosis), respectively. Following the extraction of radiomic features from the ROIs, we only selected those radiomic features with a high correlation (Intraclass correlation coefficient >0.8).

### 2.5 Radiomic feature selection and Rad-score calculation

The radiomic features were screened using Python (version 3.2). Initially, missing values were imputed using the median value of their respective feature types, and features with zero variance were removed from the dataset. Subsequently, the feature dataset was standardized using zero-mean normalization. The LASSO regression model was used to select the most discriminative radiomic features, with parameter tuning conducted through 10-fold cross-validation. The Rad-score is a linear combination of LASSO-selected features weighted by their regression coefficients. The Rad-score for each IA was derived using an radiomic scoring algorithm (Equation 1).


(1) $$\mathrm{Rad}-\mathrm{score}=\left(\sum_{1}^{i}\beta_{i}X_{i}+\beta_{0}\right)\times 100$$

${Χ}_{i}$ is the $i^{th}$ selected radiomic features and $\beta_{i}$ is the LASSO regression coefficient for that feature, intercept = 0.026.

### 2.6 Modeling and validation

A predictive model for IA rupture was developed using the Rad-score based on optimal radiomic features derived from HR-VWI. The predictive accuracy of the model was assessed using receiver operating characteristic (ROC) curves. Concurrently, these optimal features were integrated into three ML algorithms, XGBoost, LightGBM, and CART, to construct predictive models for IA rupture from HR-VWI radiomic images. The three ML models are non-linear ensembles that iteratively optimize feature interactions.

The predictive performance of the ML models was evaluated using ROC curves, and the best model was selected based on these analyses. The predictive efficacy of each ML model was quantified using metrics, including ROC, area under the curve (AUC), sensitivity, specificity, accuracy, positive predictive value (PPV), and negative predictive value (NPV). Calibration curves were used to assess the concordance between predicted probabilities and observed outcomes for the best ML and Rad-score models. Decision curve analysis (DCA) was employed to compare the net clinical benefits of the optimal ML and Rad-score models.

### 2.7 Statistical analysis

In this study, quantitative data are presented as mean ± standard deviation or median (interquartile range), and categorical data are expressed as counts (percentages). The predictive performance of the Rad-score and three ML models was evaluated using ROC curves. A *p* < 0.05 was considered statistically significant. The Rad-score cut-off for IA rupture was determined using the Youden index. The data were statistically analyzed, and images were graphically represented using R software (version 4.2.3).

## 3 Results

### 3.1 Patient characteristics

The clinical and radiological characteristics of the patients in the training, test, and external validation cohorts are summarized in Table 1. Significant differences were observed in the morphological features of ruptured and unruptured IAs, including irregular shape (*p* < 0.001 in the training cohort, *p* = 0.030 in the test cohort) and the presence of daughter aneurysms (*p* < 0.001 in the training cohort, *p* = 0.011 in the test cohort). Additionally, the anatomical locations of IAs showed significant variations across cohorts (*p* = 0.001 in the training cohort, p < 0.001 in the test cohort, and *p* = 0.004 in the external validation cohort).

**Table 1:** Clinical and radiological characteristics of enrolled patients.

Characteristics	Cohorts								
	Training (*n* = 284)			Test (*n* = 72)			External Validation (*n* = 66)		
	Ruptured (*n* = 64)	Unruptured (*n* = 220)	*p* value	Rupture(*n* = 25)	Unruptured (*n* = 47)	*p* value	Ruptured (*n* = 27)	Unruptured (*n* = 39)	*p* value
I. Clinical Characteristics									
Age, mean ± SD (years)	58.0 ± 10.4	60.2 ± 10.3	0.132	57.4 ± 12.0	57.1 ± 10.7	0.719	58.3 ± 7.7	61.3 ± 11.0	0.232
Gender (%)			0.721			0.989			0.534
Male	21 (32.8)	67 (30.5)		9 (36.0)	17 (36.2)		11 (40.7)	7 (18.0)	
Female	43 (67.2)	153 (69.5)		16 (64.0)	30 (63.8)		16 (59.3)	32 (82.0)	
Previous SAH history (%)	0 (0)	2 (0.9%)	0.446	1 (4.0)	1 (2.1)	0.651	1 (3.7)	1 (2.6)	0.794
Hypertension (%)	35 (54.7)	118 (53.6)	0.882	12 (48.0)	20 (42.6)	0.663	6 (22.2)	31 (79.5)	0.481
Hyperlipemia (%)	22 (34.4)	77 (35.0)	0.927	11 (44.0)	18 (38.3)	0.644	12 (44.4)	14 (35.9)	0.492
CAD (%)	3 (4.7)	24 (10.9)	0.136	1 (4.0)	5 (10.7)	0.339	1 (3.7)	7 (18.0)	0.084
Diabetes mellitus (%)	4 (6.3)	28 (12.7)	0.150	0 (0)	2 (4.3)	0.302	3 (11.1)	6 (15.4)	0.625
History of smoking (%)	14 (21.9)	48 (21.8)	0.992	6 (24.0)	13 (27.7)	0.742	6 (22.2)	10 (25.6)	0.534
II. Morphological characteristics of aneurysm									
Irregular aneurysm shape	44 (68.8)	61 (27.7)	<0.001	14 (56.0)	14 (29.8)	0.030	16 (59.3)	14 (35.9)	0.062
Daughter Aneurysm	26 (40.6)	24 (10.9)	<0.001	7 (28.0)	3 (6.4)	0.011	5 (18.5)	4 (10.3)	0.344
Position			0.001			<0.001			0.004
Internal carotid artery	22 (34.4)	125 (56.8)		6 (24.0)	30 (63.8)		4 (14.8)	20 (51.3)	
Anterior cerebral artery	6 (9.4)	9 (4.1)		1 (4.0)	5 (10.7)		5 (18.5)	4 (10.3)	
Anterior communicating artery	7 (10.9)	13 (5.9)		5 (20.0)	2 (4.3)		6 (22.2)	1 (2.6)	
Middle cerebral artery	10 (15.6)	41 (18.6)		4 (16.0)	8 (17.0)		4 (14.8)	9 (23.1)	
Posterior circulation	5 (7.8)	18 (8.2)		4 (16.0)	1 (2.1)		5 (18.5)	0 (0)	
Posterior communicating artery	14 (21.9)	14 (6.4)		5 (20.0)	1 (2.1)		3 (11.1)	5 (12.8)	
Lateral wall	49 (76.6)	183 (83.2)	0.230	19 (76.0)	43 (91.5)	0.072	20 (74.1)	32 (82.1)	0.443
Bifurcation	15 (23.4)	37 (16.8)		6 (24.0)	4 (8.5)		7 (25.9)	7 (17.9)	

SD, standard deviation, CAD, coronary artery disease.

### 3.2 Radiomic feature extraction and optimal radiomic features selection

We automatically extracted 107 radiomic features from each ROI in the IA and PA walls of the HR-VWI images, resulting a total of 214 distinct features. The detailed results of these features were categorized into seven classes (Table 2).

**Table 2:** Types and quantities of radiomic features with IAs and parent arteries.

Feature category	Abbreviation	Quantity
First-order statistics	/	36
Shape	/	28
Gray-level co-occurrence matrix	GLCM	48
Gray-level dependence matrix	GLDM	28
Gray-level run length matrix	GLRLM	32
Gray-level size zone matrix	GLSZM	10
Neighboring gray-tone difference matrix	NGTDM	10

**Figure 4:**
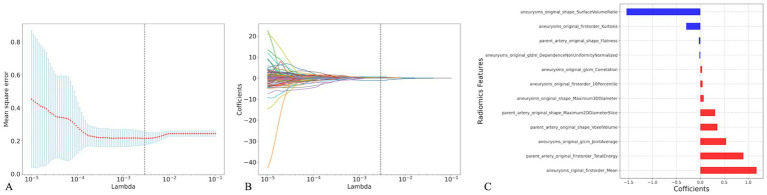
Radiomics feature selection based on the least absolute shrinkage and selection operator (LASSO). Plot of LASSO coefficients-lambda **(A)**, 10-fold cross-validation via minimum criteria was used in the LASSO model. The LASSO coefficient profiles of the radiomic features **(B)**, Coefficients of the 12 optimal radiomics features**(C)**.

One-way analysis of 214 radiomic features from the training cohort identified 138 significant features (*p* < 0.05). Figure 4 presents the optimal features selected using the least absolute shrinkage and selection operator (LASSO) regression, with parameter tuning conducted using 10-fold cross-validation to minimize overfitting. Post-regression, eight IA and four PA wall features were identified as optimal for the IA risk association (Figure 4, Table 3).

**Table 3:** Categories, names, coefficients and interpretation of optimal radiomics features.

Category	Name	Coefficient	Interpretation
Aneurysm			
Shape features	Maximum 3D diameter	0.069642	(+) Larger size → Higher risk
	Surface volume ratio	−1.574122	(−) Complex morphology → Lower risk
First-order statistics	10 Percentile	0.048111	(+) Lower calcification/thrombus → Higher risk
	Kurtosis	−0.295855	(−) Homogeneous intensity → Lower risk
	Mean	1.174627	(+) Elevated intensity → Inflammation/edema
GLCM	Correlation	0.035542	(+) Ordered texture → Mechanical stability
	Joint average	0.537752	(+) Uniform composition → Lower risk
GLDM	Dependence non-uniformity normalized	−0.000202	(−) Uniform dependencies → Lower risk
Parent Artery			
Shape features	Flatness	−0.000314	(−) Spherical shape → Uniform stress distribution
	Maximum 2D diameter slice	0.306635	(+) Focal enlargement → Higher hemodynamic stress
First-order statistics	Voxel volume	0.355955	(+) Enlarged parent artery → Vascular remodeling
	Total energy	0.898951	(+) Signal heterogeneity → Inflammatory activity

GLCM, Gray-level co-occurrence matrix; GLDM, Gray-level dependence matrix.

The analysis revealed that eight radiomic features were positively correlated with IA rupture risk, and four were inversely correlated. Of these, Surface Volume Ratio, Mean, and Total Energy contributed most significantly to the Rad-score predictive model.

### 3.3 Rad-score predictive modeling and evaluation

The Rad-score for each IA in the training cohort was calculated using 12 optimal radiomic features, yielding an AUC, sensitivity, specificity, and accuracy of 0.858 [95% confidence interval (CI): 0.804–0.911], 71.3, 88.4, and 80.9%, respectively. In the test cohort, the model demonstrated an AUC, sensitivity, specificity, and accuracy of 0.800 (95% CI: 0.726–0.875), 73.3, 83.5, and 79.6%, respectively. The external validation cohort results yielded an AUC, sensitivity, specificity, and accuracy of 0.770 (95% CI: 0.720–0.819), 69.2, 76.4, and 73.3%, respectively. Table 4 presents the detailed outcomes of this comparison.

**Table 4:** The performance of four constructed models to predict IA risk in training, test, and external validation cohorts.

Cohorts	Model	AUC	Cut-off	Sens	Spec	Acc	PPV	NPV	F1-score
Training	XGBoost	0.983 (0.970–0.996)	0.412 (0.377–0.462)	0.954 (0.910–0.998)	0.938 (0.893–0.982)	0.945 (0.944–0.945)	0.922 (0.867–0.978)	0.963 (0.928–0.999)	0.938 (0.888–0.988)
	LightGBM	0.916 (0.880–0.952)	0.471 (0.446–0.523)	0.793 (0.708–0.878)	0.884 (0.825–0.943)	0.844 (0.843–0.846)	0.841 (0.762–0.921)	0.846 (0.781–0.912)	0.816 (0.734–0.899)
	CART	0.909 (0.868–0.951)	0.499 (0.451–0.545)	0.805 (0.721–0.888)	0.857 (0.792–0.922)	0.834 (0.833–0.836)	0.814 (0.732–0.896)	0.850 (0.784–0.915)	0.812 (0.726–0.892)
	Rad-score	0.858 (0.804–0.911)	52.7 (47.8–59.2)	0.713 (0.618–0.808)	0.884 (0.825–0.943)	0.809 (0.808–0.811)	0.827 (0.741–0.912)	0.798 (0.728–0.869)	0.766 (0.674–0.857)
Test	XGBoost	0.891 (0.856–0.927)	0.409 (0.360–0.454)	0.837 (0.777–0.896)	0.852 (0.803–0.900)	0.846 (0.845–0.846)	0.799 (0.735–0.862)	0.881 (0.837–0.926)	0.818 (0.755–0.879)
	LightGBM	0.870 (0.833–0.907)	0.497 (0.447–0.533)	0.694 (0.619–0.768)	0.890 (0.848–0.932)	0.809 (0.808–0.810)	0.816 (0.748–0.884)	0.805 (0.754–0.856)	0.750 (0.677–0.822)
	CART	0.834 (0.789–0.878)	0.499 (0.451–0.539)	0.735 (0.663–0.806)	0.837 (0.787–0.887)	0.795 (0.794–0.796)	0.761 (0.690–0.831)	0.818 (0.766–0.869)	0.748 (0.676–0.818)
	Rad-score	0.800 (0.726–0.875)	42.1 (39.9–48.6)	0.733 (0.621–0.845)	0.835 (0.761–0.909)	0.796 (0.794–0.798)	0.733 (0.621–0.845)	0.835 (0.761–0.909)	0.733 (0.621–0.845)
External Validation	XGBoost	0.864 (0.825–0.903)	0.409 (0.361–0.453)	0.82 (0.729–0.896)	0.840 (0.790–0.889)	0.903 (0.819–0.820)	0.787 (0.787–0.851)	0.844 (0.795–0.893)	0.789 (0.726–0.853)
	LightGBM	0.841 (0.801–0.881)	0.46 (0.409–0.486)	0.679 (0.607–0.752)	0.840 (0.790–0.889)	0.771 (0.770–0.772)	0.761 (0.690–0.831)	0.777 (0.723–0.831)	0.718 (0.646–0.79)
	CART	0.803 (0.756–0.849)	0.499 (0.451–0.538)	0.679 (0.607–0.752)	0.825 (0.774–0.877)	0.763 (0.762–0.764)	0.745 (0.674–0.816)	0.774 (0.720–0.829)	0.71 (0.639–0.783)
	Rad-score	0.770 (0.720–0.819)	43.4 (41.3–48.8)	0.692 (0.620–0.764)	0.764 (0.707–0.821)	0.733 (0.732–0.734)	0.688 (0.616–0.759)	0.768 (0.711–0.825)	0.69 (0.618–0.761)

IA, intracranial aneurysm; AUC, area under the receiver operating characteristic curve; Sen, sensitivity; Spe, specificity; Acc, accuracy; PPV, positive predictive value; NPV, negative predictive value.

### 3.4 ML predictive modeling and evaluation

**Figure 5:**
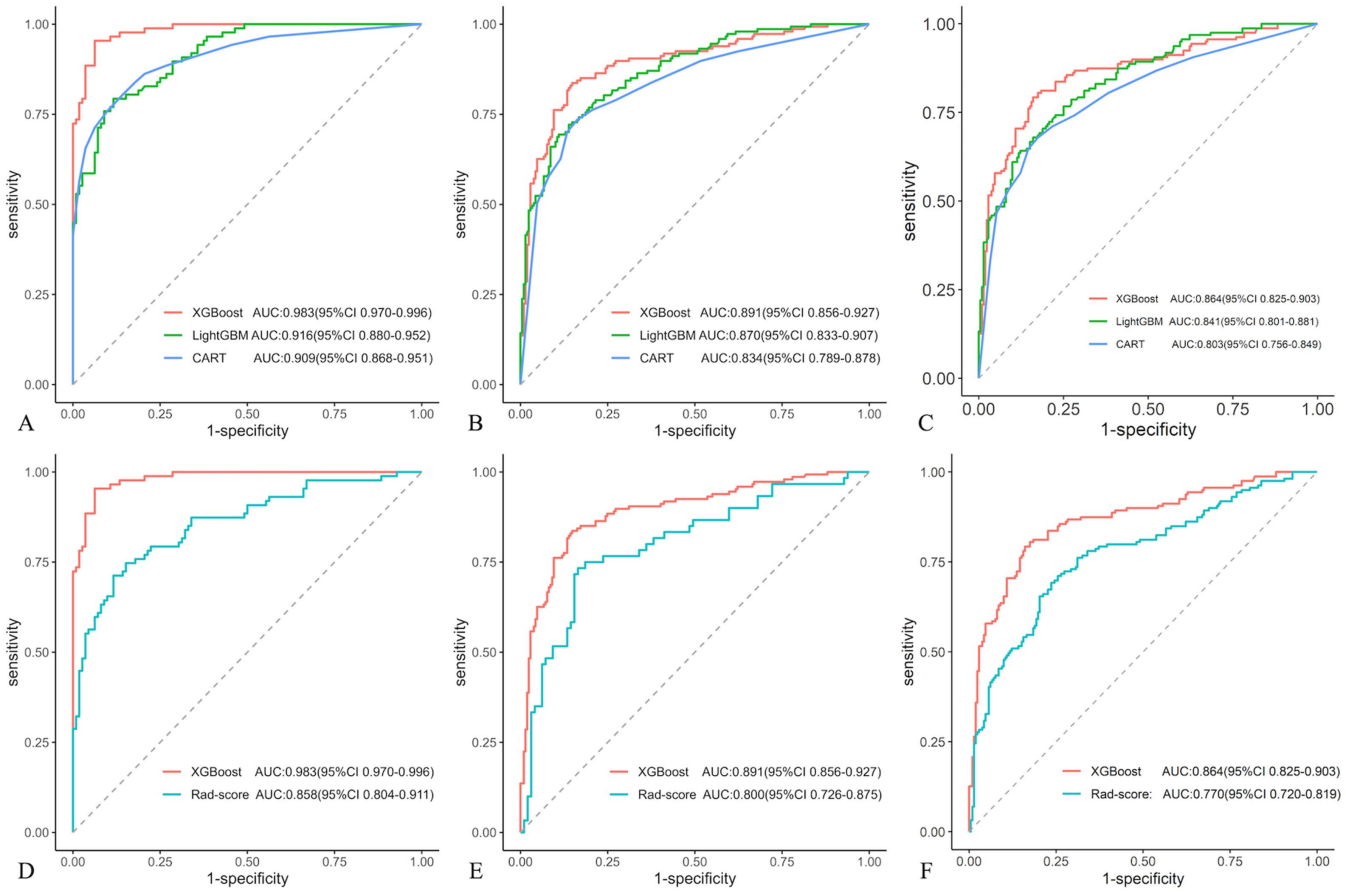
Receiver operating characteristic (ROC) curves compared in three machine learning (ML) models in different cohorts: training **(A)**, test **(B)**, and external validation **(C)**. And Receiver operating characteristic (ROC) curves displayed in XGBoost and Rad-score models in different cohorts: training **(D)**, test **(E)**, and external validation **(F)**.

In the training cohort, 12 optimal radiomic features were employed to construct IA rupture prediction models using the XGBoost, LightGBM, and CART algorithms. After 10-fold cross-validation and model refinement, the XGBoost model achieved the highest AUC values with sensitivity, specificity, and accuracy of 95.4, 93.8, and 94.5%, respectively. In the test cohort, the XGBoost model maintained the highest AUC, with sensitivity, specificity, and accuracy of 83.7, 85.2, and 84.6%, respectively. In the external validation cohort, the XGBoost model exhibited the highest AUC, with sensitivity, specificity, and accuracy of 82.0, 84.0, and 90.3%, respectively (Table 4, Figure 5).

### 3.5 Comparison of the XGBoost and Rad-score models

**Figure 6:**
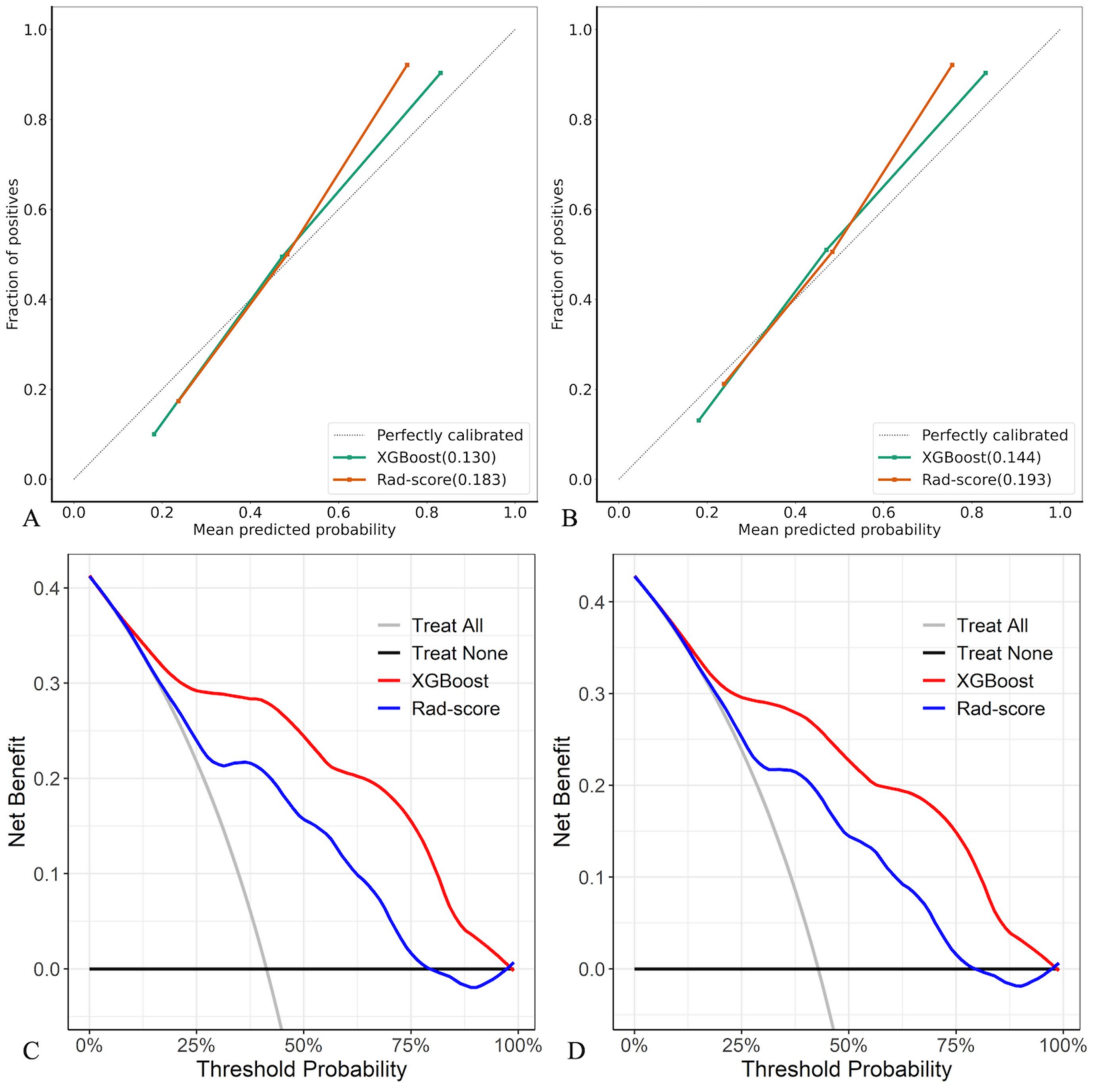
Calibration curves and DCA of XGBoost and Rad-score models in test **(A,C)** and external validation **(B,D)** cohorts.

Compared with the rad-score model, the XGBoost model also achieved higher AUC values (Table 4, Figure 5). The DeLong test revealed statistically significant AUC differences between the XGBoost and Rad-score models in the test and external validation cohorts (*p* < 0.05). Calibration curves for the test cohort presented Brier scores of 0.130 for XGBoost and 0.183 for Rad-score, while the external validation cohort scores were 0.144 and 0.193, respectively (Figure 6). DCA indicated that the XGBoost model provided a greater net clinical benefit than the Rad-score model (Figure 6).

## 4 Discussion

In this study, HR-VWI was employed for the initial extraction of radiomic features from IAs and their PAs, and 12 key radiomic features were successfully identified. Rad-score and ML models were developed independently of patient clinical characteristics and conventional morphological features of IAs. Both models were effective in predicting the risk of IA rupture, with the ML model, particularly the one using the XGBoost algorithm, exhibiting enhanced predictive accuracy, including improved differentiation, calibration, and clinical applicability. These findings indicate that the XGBoost model could provide an innovative, non-invasive, and radiation-free approach for IA rupture risk assessment.

Previous radiomic studies predicting IA rupture risk have predominantly used DSA and CTA imaging, each with inherent limitations, including the invasiveness of DSA and radiation exposure associated with CTA (Chen et al., 2022; Beaman et al., 2023). This study pioneered the application of non-invasive, radiation-free HR-VWI for radiomic feature extraction, offering a significant advancement over traditional methods. From a comprehensive set of 214 features, 12 were identified as optimal: 8 from the IA walls and 4 from the PA walls. The surface-area-to-volume ratio among the IA wall features exhibited the greatest impact, signifying the geometric relationship between the aneurysm surface area and volume. A lower ratio indicates a smoother IA wall, potentially reducing the shear force and associated rupture risk, thereby highlighting the critical link between IA shape and rupture propensity. This is consistent with the findings of Liu et al. (2021) using DSA image analysis, where the surface-area-to-volume ratio and IA wall smoothness were key predictors. Furthermore, gray-level co-occurrence matrix (GLCM) correlation features, denoting the linear relationship between gray values in IA wall images, were selected as the optimal features. These features, along with others reflecting the distribution of high gray values, provide nuanced insights into IA wall characteristics.

Previous studies have suggested that 3D radiomics features may provide a more comprehensive characterization of aneurysms. However, 2D and 3D radiomics features inherently capture different spatial information. Additionally, 3D features are sensitive to anisotropic voxel resolution and require substantial computational resources for segmentation and feature extraction. Therefore, in this study, the target area was delineated by selecting the slice with the largest aneurysm diameter.

Building on recent research suggesting a correlation between HR-VWI enhancement within 3 mm of the IA neck and saccular aneurysm development (Samaniego et al., 2020), this study incorporated PA wall imaging features. The total energy from first-order statistics, indicating the overall gray-scale intensity, emerged as the most influential PA wall feature. Besides, IA wall flatness, maximum 2D diameter, and voxel volume were identified as the optimal features. These radiomic features provide a multidimensional profile of IAs and PAs, encompassing size, shape, gray-scale distribution within the ROI, and linear correlations.

Rad-score, a composite radiomic feature score, encapsulates a multitude of radiomic features into a single, biologically meaningful, and clinically relevant metric (van Timmeren et al., 2020). Tong et al. (2021) conducted a landmark multicenter study using DSA images, examined 105 cases of multiple IAs, and demonstrated the superiority of the Rad-score over traditional morphological models in predicting IA rupture. This study extended the evaluation of the relationship between HR-VWI radiomic features and the risk of IA rupture by focusing on rupture risk and excluding patient clinical characteristics or conventional morphological features. In our study, the Rad-score was calculated using 12 optimal features from both the IA and PA walls derived from HR-VWI images. The resulting multidimensional Rad-score model, which integrated HR-VWI heterogeneity, yielded an AUC of 0.800 in the test cohort (sensitivity, 73.3%; specificity, 83.5%; accuracy, 79.6%) and 0.770 in the external validation cohort (sensitivity, 69.2%; specificity, 76.4%; and accuracy, 73.3%). These results suggest that the Rad-score can quantitatively capture imaging heterogeneity and offer a valuable imaging biomarker for IA rupture risk assessment.

The Rad-score model, developed using HR-VWI radiomics, demonstrated promising predictive capabilities. However, there is scope for further enhancement. Yang et al. (2023) demonstrated that integrating CTA radiomic features with ML models could significantly enhance the prediction of IA rupture, with an AUC of 0.918 following extensive cross-training. This underscores the potential of ML algorithms to unravel complex variable interactions within large datasets, thereby facilitating the identification of novel model configurations or training methodologies to enhance predictive accuracy.

In this study, 12 optimal radiomic features derived from HR-VWI were incorporated into three ML models, followed by rigorous training and 10-fold cross-validation, revealing that the XGBoost model was the most effective. In the training cohort, the AUC was 0.983, with a sensitivity, specificity, and accuracy of 95.4, 93.8, and 94.5%, respectively. These metrics were slightly reduced but still impressive in the test cohort, with an AUC, sensitivity, specificity, and accuracy of 0.891, 83.7, 85.2, and 84.6%, respectively. Similarly, the external validation cohort exhibited an AUC, sensitivity, specificity, and accuracy of 0.864, 82.0, 84.0, and 90.3%, respectively. The XGBoost model demonstrated high stability and generalization, and significantly outperformed the Rad-score model in predictive efficacy (Delong test, *p* < 0.05), including calibration and clinical net benefit.

Radiomic-based ML has shown promising accuracy in early identification of IA rupture status or prediction of rupture risk, revealing its potential application in clinical practice (Daga et al., 2024; Zhong et al., 2024). XGBoost, an efficient and novel boosting algorithm optimized from fundamental ML techniques, has advantages such as high training efficiency, effective predictive power, adjustable parameters, and user-friendliness (Wang et al., 2022). It has been successfully applied in various medical imaging contexts, including diagnosing lung nodules (Nishio et al., 2018), risk assessment in coronary CTA (van Rosendael et al., 2018), and predicting acute strokes in magnetic resonance perfusion imaging (Livne et al., 2018). This study marks the inaugural application of the XGBoost algorithm to HR-VWI radiomic data, yielding a satisfactory predictive accuracy for IA rupture risk. Exploration of ML algorithms, particularly XGBoost, in the diagnosis, risk assessment, and predictive modeling of IAs is warranted and holds considerable promise.

Our study focused on HR-VWI radiomic features for predicting IA rupture risk. To isolate the independent predictive power of these features, we eliminated confounding clinical factors and subjectivity-prone aneurysm morphological features. Instead, we concentrated on radiomic features of the IA wall and PA wall. By doing so, we aimed to establish a baseline reference for future multimodal integration, while acknowledging that established clinical and morphological factors, such as aneurysm multiplicity, family history, smoking, and hypertension, also significantly contribute to rupture risk (Thompson et al., 2015).

This study has several limitations. First, the retrospective design may introduce selection bias, potentially compromising the generalizability of the conclusions. Second, the limited external validation cohort restricts robust verification of the model’s generalizability. Third, the radiomic methodology faces some constraints: two-dimensional cross-sectional analysis fails to fully characterize three-dimensional morphological heterogeneity, while potential contrast extravasation in ruptured aneurysms may distort feature stability and subjective variations inherent in manual target delineation may reduce result reproducibility, particularly due to inter-observer differences in boundary definition. Finally, the lack of standardized cross-institutional imaging protocols and unified feature definitions reduces result reproducibility. Future work should involve prospective multicenter studies integrating three-dimensional whole-lesion segmentation techniques and establishing standardized imaging processing criteria to systematically enhance model performance.

## 5 Conclusion

Radiomic analysis of HR-VWI images successfully identified optimal features to predict IA rupture. The Rad-score model, developed using these HR-VWI-derived features, demonstrated effective predictive accuracy for IA rupture. Furthermore, the integration of these features with the XGBoost ML algorithm produced results that significantly exceeded those of the Rad-score model. This ML methodology has emerged as an invaluable tool for clinically identifying patients at a high risk of IA rupture.

## Data Availability

The raw data supporting the conclusions of this article will be made available by the authors, without undue reservation.
