# Supplementary material for: High-resolution vessel wall imaging-driven radiomic analysis for the precision prediction of intracranial aneurysm rupture risk: a promising approach
Source: Front Neurosci. 2025 Apr 22;19:1581373. doi: 10.3389/fnins.2025.1581373 (PMC12052944; doi:10.3389/fnins.2025.1581373)
Supplement: Supplementary file 1 [file Table_1.docx]

Supplementary Table S1 Scan parameters of TOF-MRA, HR-VWI, and CE-HR-VWI.

| Parameter | TOF-MRA | HR-VWI | CE-HR-VWI |
| --- | --- | --- | --- |
| FOV, mm² | 200 × 181 | 200 × 200 | 200 × 200 |
| Slice Thickness, mm | 0.60 | 0.50 | 0.50 |
| TR/TE, ms | 21/3.45 | 800/22 | 800/22 |
| Number of Slices | 160 | 80 | 80 |
| Voxel Size, mm³ | 0.6 × 0.8 × 1.4 | 0.6 × 0.6 × 0.6 | 0.6 × 0.6 × 0.6 |
| Bandwidth, Pix/Hz | 2.0/217 | 1.54/281.5 | 1.54/281.5 |
| Acquisition Matrix | 320 × 232 | 332 × 302 | 332 × 302 |
| NEX | 1 | 1 | 1 |
| Flip Angle, ° | 18 | 90 | 90 |
| Acquisition Time, min: s | 3: 36 | 5: 12 | 5: 12 |

**Notes:** FOV, Field of View; TR, repetition time; TE, echo time; NEX, number of excitations.
